# Scan-less microscopy based on acousto-optic encoded illumination

**DOI:** 10.1515/nanoph-2023-0616

**Published:** 2024-01-02

**Authors:** Andrea Marchese, Pietro Ricci, Peter Saggau, Martí Duocastella

**Affiliations:** Department of Applied Physics, Universitat de Barcelona, Martí i Franquès, 1, 08028 Barcelona, Spain; Department of Neuroscience, Baylor College of Medicine, One Baylor Plaza, S640, 77030 Houston, TX, USA

**Keywords:** acousto-optics, optical microscopy, fast imaging, single pixel camera, illumination encoding

## Abstract

Several optical microscopy methods are now available for characterizing scientific and industrial processes at sub-micron resolution. However, they are often ill-suited for imaging rapid events. Limited by the trade-off between camera frame-rate and sensitivity, or the need for mechanical scanning, current microscopes are optimized for imaging at hundreds of frames-per-second (fps), well-below what is needed in processes such as neuronal signaling or moving parts in manufacturing lines. Here, we present a scan-less technology that allows sub-micrometric imaging at thousands of fps. It is based on combining a single-pixel camera with parallelized encoded illumination. We use two acousto-optic deflectors (AODs) placed in a Mach–Zehnder interferometer and drive them simultaneously with multiple and unique acoustic frequencies. As a result, orthogonal light stripes are obtained that interfere with the sample plane, forming a two-dimensional array of flickering spots – each with its modulation frequency. The light from the sample is collected with a single photodiode that, after spectrum analysis, allows for image reconstruction at speeds only limited by the AOD’s bandwidth and laser power. We describe the working principle of our approach, characterize its imaging performance as a function of the number of pixels – up to 400 × 400 – and characterize dynamic events at 5000 fps.

## 1 Introduction

Optical microscopy has become the tool of choice in a myriad of relevant applications such as diagnostics [1], [2], neuroscience [3], fluid dynamics [4], and industrial inspection [5]. Compared to other characterization techniques, it offers key advantages including the possibility for non-invasive imaging of dynamic events, sub-micrometric spatial resolution, and direct access to the recorded information. The most common microscopy architectures are based on cameras such as charge-coupled devices (CCDs) and complementary metal oxide semiconductors (CMOS) for image acquisition. These devices offer millions of pixels thus enabling high-quality image collection. However, the readout of all this information is time-consuming, with a typical maximum acquisition speed of some hundreds of frames per second (fps) – insufficient to characterize fast phenomena in fields as relevant as neuroscience [3], [6], biochemical analysis [7], [8], optical quality inspection [9], or plasma physics [10]. Faster cameras exist, but they typically offer decreased sensitivity (not compatible with fluorescent samples) and a significantly higher price. Note that cameras are already one of the most expensive components of a microscope. Alternatively, it is possible to implement microscopy systems using single-pixel detectors that feature a very short response time at the sub-nanosecond time scale, high sensitivity and a wide spectral range, from ultraviolet to infrared detection [11]. Nevertheless, because all photons arriving from different positions within a sample are collected at the same pixel, the spatial information is lost. Therefore, to reconstruct an image, additional steps are necessary. The traditional way is to scan a laser beam across the sample point by point, as in confocal microscopy [12] and two-photon microscopy [13], [14]. Such a sequential approach is typically time-consuming, with a minimum pixel dwell time required at each sample position plus the time required for the scanner to move. As a result, current scanning systems offer imaging speeds similar to those of cameras [6].

A promising solution to increase imaging speed consists of using single-pixel detectors with encoded illumination. The overall idea is to simultaneously illuminate different regions of the sample using specific encoding sequences. By knowing *a priori* such sequences, all the information collected from the single pixel detector can be decoded and an image can be reconstructed. Still, the most common implementations rely on mechanical moving parts, such as spinning masks [15], [16] or Hadamard illumination [17]. More advanced approaches include the use of broadband pulsed lasers, a diffraction grating and a virtually imaged phased array [18], [19], or compressed ultrafast photography [20], [21]. While striking imaging rates of millions or even billions of frames per second can be achieved, these systems can be difficult to implement in practice, often require complex computational processing, and can be limited by the spectral response of the sample. An encouraging alternative is using digitally synthesized frequency beats. In this case, acousto-optic deflectors (AODs) are placed on a Mach–Zehnder interferometer to generate a line of overlapping spots, each with a unique beat frequency [22]. Unfortunately, retrieving a full 2D image requires a scanning mirror to translate the encoded line pattern [23], [24]. This adds complexity to the overall system, with the need for synchronization between illumination encoding and mechanical scanning. More importantly, a fundamental limit on the signal integration time persists. Indeed, increasing the scanning velocity comes at the cost of reduced pixel dwell time. This can have devastating consequences for fluorescent imaging, where the fluorescent lifetime of the molecules (tens of nanoseconds [25]) needs to be considered. Note that, if the pixel-dwell time is reduced below the fluorescence lifetime, as it can occur for high-speed scanning, part of the fluorescence photons do not contribute to image contrast, degrading signal-to-noise ratio (SNR) and inducing possible pixel crosstalk. In addition, due to fluorophore saturation, increasing the excitation power to compensate for the signal loss is ineffective. A solution to this problem is to obviate any scanning system by directly performing full-field illumination encoding. In this case, the effective pixel dwell time can be increased by a factor proportional to the number of pixels of the image – several orders of magnitude. A step in this direction is a recently proposed system based on exploiting the interference between two frequency comb lasers [26]. However, the system lacks encoding adjustability and requires synchronization between two different dual-comb systems, a potentially challenging task.

Here, we propose a new scan-less microscope architecture that allows for fast full-field imaging and adjustable spatiotemporal resolution. Our design, termed frequency-encoded microscope (FREMIC), is based on generating arrays of orthogonal light stripes with two AODs and two cylindrical lenses, each placed at a different arm of the Mach–Zehnder interferometer. At the sample plane, interference between the light stripes results in flickering spots, each with a unique and known intensity modulation frequency. By using a single-pixel detector, all the information provided from the sample is merged, but *a priori* knowledge of the frequency-position encoding allows the image reconstruction. Notably, we can control the position of the spots, the field-of-view (FOV), and the number of image pixels by simply adjusting the radiofrequency signal that drives the AODs. Such electronic control of the illumination allows for very rapid adjustability of the image properties, down to tens of microseconds, without any limitation due to the fixed response time of mechanical mirrors. In experiments herein, we perform a detailed characterization of the optical performance of our encoded microscope and demonstrate its feasibility by imaging a dynamic system at rates up to 5 kHz.

## 2 Principle and design of FREMIC

The general principle of FREMIC, as in any encoded illumination system, is to map univocally the different spatial coordinates (*x*, *y*) of a sample onto a specific optical code. In particular, FREMIC uses frequency encoding, based on illuminating each coordinate with light whose intensity is modulated over time at a unique and distinct frequency *f*(*x*,*y*). Thus, the relationship between spatial coordinates and frequency can be written as:

(1) (x,y)↔f(x,y)

**Figure 1:**
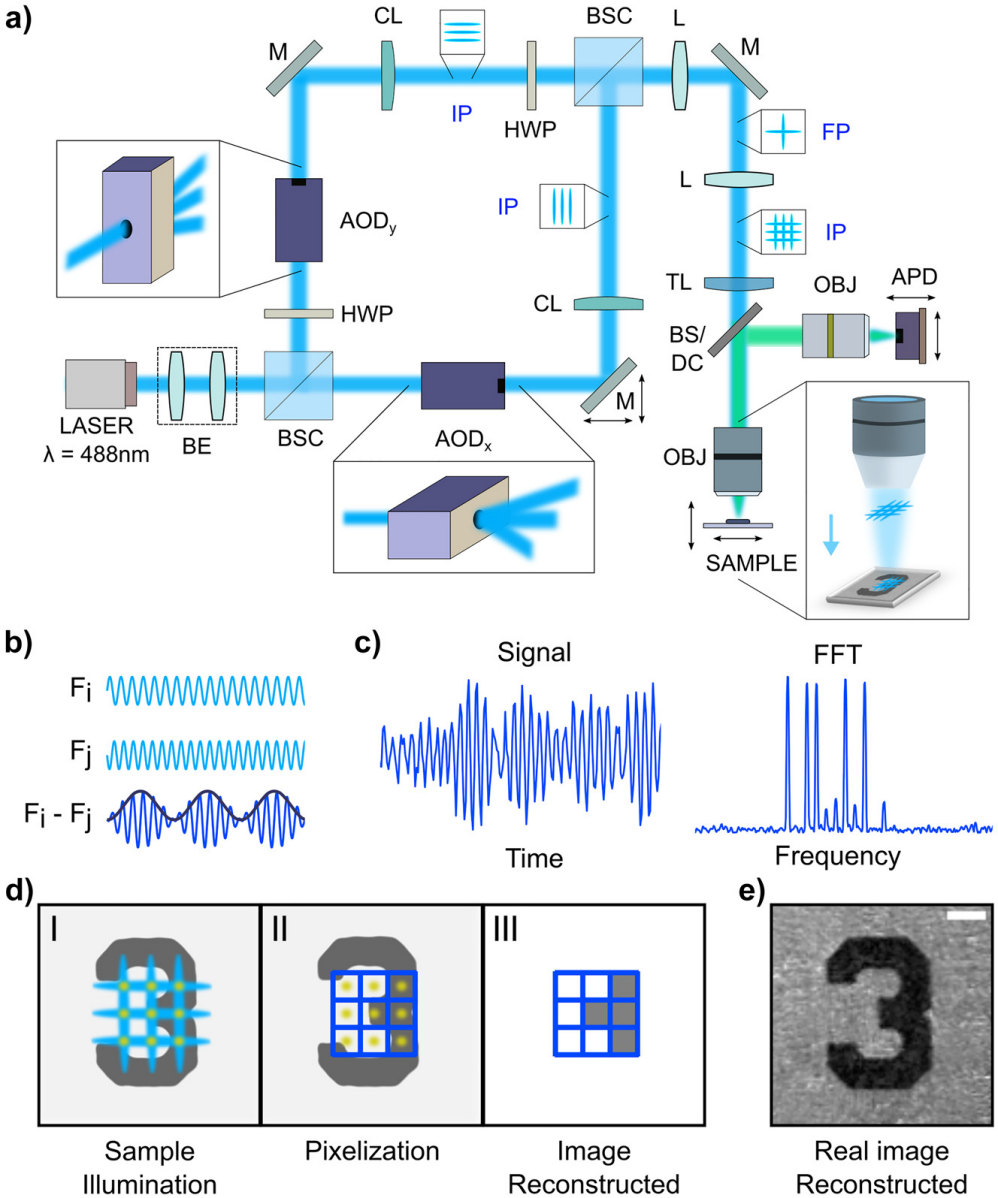
Principle and implementation of FREMIC. (a) Schematic of experimental optical setup. Insets show transverse light profiles along the optical path, in specific conjugate image-planes (IP) and Fourier-planes (FP). BE: beam expander; HWP: half-wave plate; BSC: beam splitter cube; AOD: acousto-optic deflector; CL: cylindrical lens; M: mirrors; L: relay lens; TL: tube lens; OBJ: objective; BS/DC: beam splitter/dichroic mirror; APD: avalanche photodiode. (b) Illumination encoding is performed by illuminating the sample with a grid of crossing light stripes, each with a different frequency. At the intersection point between two such light stripes, with frequencies *F*_*i*_ and *F*_*j*_, frequency beats with value *F*_*i*_−*F*_*j*_ are obtained. (c) Left: temporal fingerprint of the signal collected by the APD where the beats from multiple intersecting stripes are collected. Right: corresponding spectral decomposition obtained by performing a Fourier transform. (d) Scheme of the image reconstruction process. I: the sample is illuminated with a grid of light stripes; II: from the signal spectrum, the amplitude peaks corresponding to the beat frequencies of the crossing points are extracted; III: the mapping between frequency values and spatial coordinates is performed. (e) Example of a reconstructed image of 100 × 100 pixels taken from a USAF target. Scale bar 10 μm.

To implement the bijective function described in Eq. (1), FREMIC uses an original architecture based on a Mach–Zehnder interferometer, as shown in Figure 1a. The key components of the system are two AODs, each one placed at a different arm of the interferometer. By simultaneously driving the AODs with *N* different radio frequency signals, each with a unique frequency *f*_*i*_, light is diffracted into a fan of *N* beamlets. Importantly, due to the nature of the acousto-optic effect, the beamlets are not only deflected at a specific angle, but they are also frequency-shifted. Specifically, each beamlet carries a unique frequency given by *F*_*i*_ = *ν*_*L*_ + *f*_*i*_, where *ν*_*L*_ is the frequency of the incident laser beam [27]. Another distinct feature of FREMIC is that the AODs are orthogonally oriented, and so is the direction of the fan of beamlets in each arm of the interferometer. By placing a cylindrical lens (CL) at the output of each AOD, the two fans of beamlets can be converted into two arrays of *N* light stripes, each orthogonal to one other. Once they meet after the interferometer, they form an *N* × *N* grid of crossing light stripes. Notably, at each intersection point, there is an overlap of two coherent beams with slightly different frequencies, giving rise to the phenomenon of beats. Thus, when a beam with frequency *F*_*i*_ overlaps with a beam of frequency *F*_*j*_ at position (*x_i_*, *y_i_*), they interfere resulting in a flickering spot whose intensity *M*_*i*,*j*_(*t*) is given by:

(2) $$M_{i,j}\left(t\right)=m_{i,j}\cdot \sin\left(2\pi F_{i,j}t\right)$$

where *m*_*i*,*j*_ is the interference amplitude and *F*_*i*,*j*_ = *F*_*i*_ − *F*_*j*_ = *f*_*i*_ − *f*_*j*_ is the beat frequency (Figure 1b). Note that the flickering frequency only depends on the driving AOD frequencies [28]. Therefore, by properly choosing the *N* frequencies on each AOD, it is possible to generate an array of *N* × *N* flickering spots, each with a unique modulation frequency and spatial position (see Supporting Information, Section S1). This effectively constitutes the encoding relationship described in Eq. (1). The 2D grid of flickering spots can be projected on the sample using relay optics, and the corresponding signal can be collected using a single-pixel detector. In this case, the signal depends on the local sample response *s*(*x*,*y*) and point spread function (PSF) of the imaging system. Considering the sample to reflect or re-emit light faster than the highest light modulation frequency – a fair assumption, except when using molecules with a long fluorescent lifetime [29] – *s*(*x*,*y*) will be precisely modulated by *M*_*i*,*j*_(*t*) at (*x_i_*, *y_i_*). Thus, the light intensity collected by the single-pixel camera when illuminating an area A will contain a superposition of all the flickering frequencies within that sample region, which can be written as (Figure 1c, left):

(3) $$I\left(t\right)=\sum_{i,j=1}^{N}\int_{A}s\left(x,y\right)\cdot M_{i,j}\left(t\right)*PSF\left(x,y\right)dxdy$$

Given that *M*_*i*,*j*_(*t*) is known *a priori*, the value *s*(*x*,*y*) can be retrieved after applying a decoding step (Figure 1c, right) – a spectral analysis described in detail in Supporting Information (Section S8). Figure 1d shows schematically the main steps necessary to reconstruct an image of a sample containing bright and opaque parts (dark regions delimiting the number). The Fourier-transformed signal returns several peaks corresponding to the beat frequencies of the crossing points (I). Each extracted amplitude is assigned to a pixel in the final image (II). By knowing the relationship between beat frequencies and spatial position in the pattern, we can reconstruct the final image (III). Figure 1e shows a demonstration of a real reconstructed image of a USAF target.

Thanks to the intrinsic high speed of the single-pixel camera used to collect the light, FREMIC allows high-rate 2D imaging. The ultimate imaging rate is given by the range of frequencies that the AODs can generate – also known as frequency bandwidth or Δ*f* – and the spectral separation between them. In detail, to be able to resolve two beat frequencies spectrally separated by *δF*, it is necessary to collect the corresponding flickering signals for a sufficient integration time *T*. This time is determined by the properties of Fourier transforms as:

(4) $$T\geq \frac{1}{\delta F}$$

Considering an *N* × *N* image, the minimum *δF* that can be obtained when driving two AODs with *N* frequencies, is (see Supporting Information, Section S1):

(5) $$\delta F=\frac{\Delta f}{N\left(N-1\right)}$$

Consequently, the minimum integration time *δt* will be:

(6) $$\delta t=\frac{1}{\delta F}=\frac{N\left(N-1\right)}{\Delta f}$$

As an example, for an acoustic bandwidth of 200 MHz, a whole 100 × 100 pixels image could be captured in about 50 µs or equivalently, at a rate of 20 kHz. For a bandwidth of 1 GHz (the state of the art for commercial AODs), a 50 × 50 pixel image could be captured in only 2.5 µs, that is, an acquisition rate of 400,000 frames per second.

Besides speed, the scan-less nature of FREMIC provides additional advantages compared to traditional point scanning systems. The first one regards the effective pixel dwell time and the phenomenon known as the fluorescent lifetime limit. To capture 100 × 100 pixel fluorescent images at a speed of 20,000 fps, a point-by-point scanning system – obviating any mechanical inertia – requires a minimum pixel dwell time of 50 µs/(100 × 100) = 5 ns. This can be shorter than the fluorescence lifetime of common dyes. Therefore, a loss of SNR and crosstalk between adjacent pixels is expected, with the consequent degradation in image quality. By performing line-by-line scanning, the problem can be partially alleviated [23], and the effective pixel dwell time can be increased by a factor equal to the number of pixels in a line – 100 in the example given. FREMIC takes a step forward in this direction and, for an acoustic bandwidth of 200 MHz, allows an increase in the effective pixel dwell time equal to the total number of pixels – 100,000 in the current example, for a total of 50 µs. This renders FREMIC a technique suitable for fast imaging of phosphorescence dyes.

Another advantage of FREMIC over a scanning system is the potential gain in SNR. Considering the total imaging time *T* to be equal in both scanning and scan-less systems, we can write (see full derivation in Supporting Information, Section S2):

(7) $$\frac{SNR_{\text{scan }-\text{ less}}}{SNR_{\text{scanning}}}=\frac{\sqrt{\left(2e\right.\;\left(RI+i_{dn}\right)\frac{N^{2}}{T}}}{\sqrt{\left(2e\right.\;\left(N^{2}\cdot RI+i_{dn}\right)\frac{1}{T}}}$$

where *e* is the electron charge, *i*_*dn*_ is the detector noise current, *R* is the detector responsivity, and *I* is the radiative power from a single spot. For a shot-noise-dominated system (*RI* ≫ *i*_*dn*_), the ratio of Eq. (7) tends to 1. In this case, the SNR of a scan-less system does not improve with respect to a scanning one. This is a common trend in multiplexing strategies [30]. Interestingly, though, for a detector-noise-dominated system (*RI* ≪ *i*_*dn*_), obviating scanning is beneficial. Indeed, the gain in SNR can scale as *N* – it would scale as $\sqrt{N}$ for line scanning systems. Given that in realistic scenarios both shot noise and detector noise are present, FREMIC is expected to outperform point and line scanning systems in terms of SNR.

## 3 Results and discussion

### 3.1 Temporal performance

**Figure 2:**
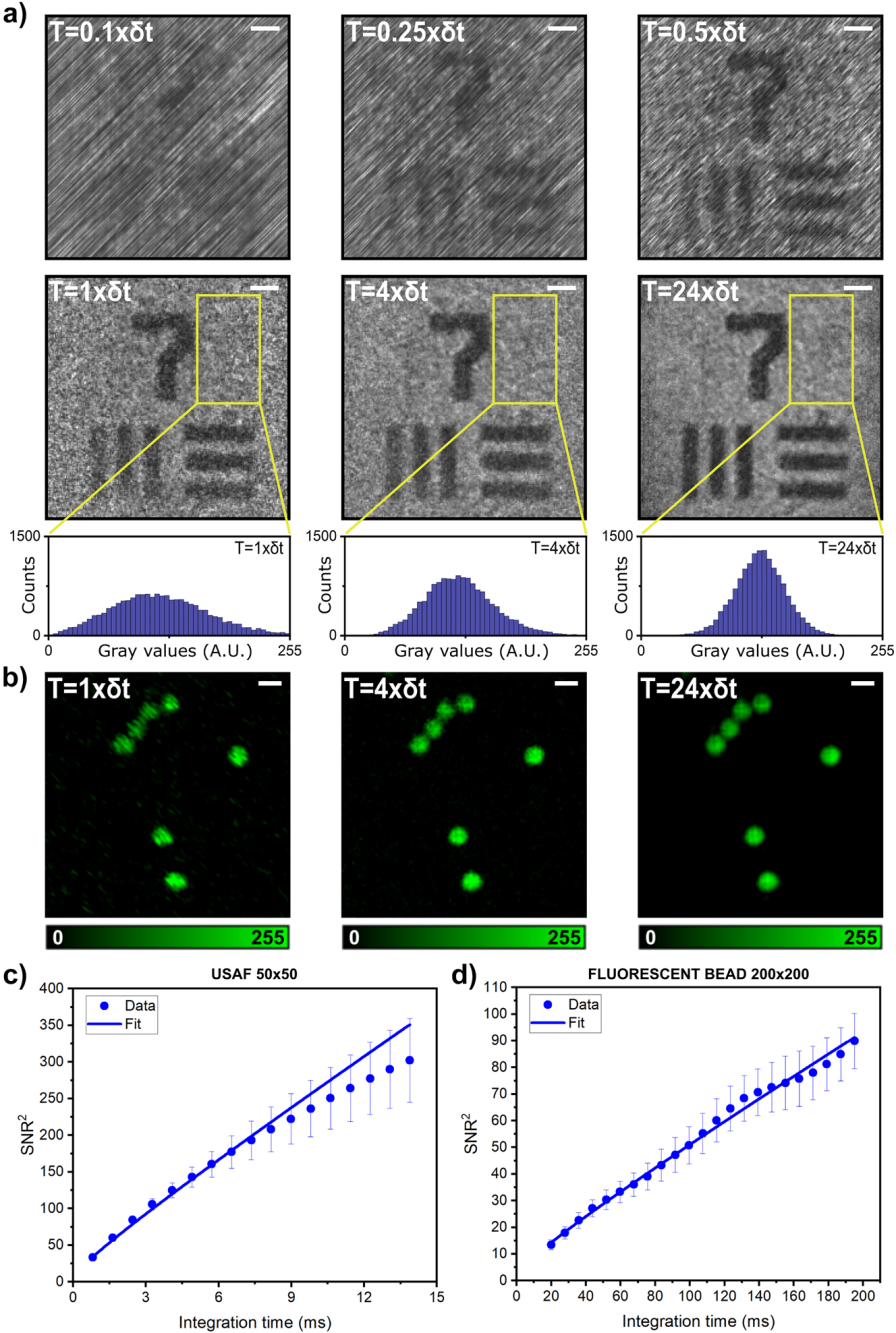
Experimental characterization of the temporal performance of FREMIC. (a) 200 × 200 pixels images acquired at *T* = 0.1 × *δt*, *T* = 0.25 × *δt*, *T* = 0.5 × *δt*, first row, and *T* = 1 × *δt*, *T* = 4 × *δt*, *T* = 24 × *δt*, second row, corresponding to conditions below and above the minimum integration time, respectively. The reflective USAF target images are normalized and visualized with the same intensity scale. Scale bar 10 µm. In the insets, the gray value distributions for *T* = 1 × *δt*, *T* = 4 × *δt* and *T* = 24 × *δt* are from the yellow areas above. (b) 200 × 200 pixels images of 4 µm diameter fluorescent beads. Scale bar 5 µm. (c) Plot of the SNR square versus integration time (from *T* = 4 × *δt* up to *T* = 68 × *δt*) calculated for an image of 50 × 50 pixels of reflective USAF target. The data and the error bars are obtained from the mean values and the standard deviation of SNR from multiple measurements, respectively. (d) Plot of the SNR square versus integration time (from *T* = 5 × *δt* up to *T* = 49 × *δt*) calculated from a fluorescent bead image of 200 × 200 pixels. The data and the error bars are obtained from the mean values and the standard deviation of SNR from multiple measurements, respectively. The least-square value fitting corresponds to (*a* + *b × t* ^1/2^)^2^, where *t* is integration time.

First, we characterized the maximum frame rate of our system for a given number of pixels. To this end, we selected a 200 × 200 pixel image which provides a good trade-off between spatial resolution and signal-to-background ratio (see the next sections for further details). Given the bandwidth of the AODs we used (Δ*f* = 12 MHz), Eq. (6) predicts a minimum integration time of *δt* = 3.3 ms, which corresponds to a frame rate of about 300 fps. As shown in Figure 2a (first-row), images of a USAF target acquired with FREMIC for integration times shorter than δt exhibit directional artifacts and an overall poor quality. In this case, we do not fulfill the conditions described in Eq. (4), and thus the frequency resolution becomes insufficient to discriminate neighboring beat frequencies. Because neighboring frequencies happen to be arranged diagonally across the image, the observed image artifacts exhibit a clear directionality (see Supporting Information, Section S1). Instead, the reconstructed images’ quality greatly improves for integration times equal to or larger than *δt*, as shown in Figure 2a (second row). Element 1 of group 7 is clearly visible, with no reconstruction artefacts present. These results confirm that experiments are in good agreement with the theoretical framework presented above. Additionally, the noise present in the images is gradually reduced by increasing the integration time. Such a trend can be better appreciated in the histogram plots from the insets below indicating the image noise gray value distribution across the highlighted yellow areas. The distribution width decreases with integration time, in agreement with the visual quality perception. The same trend occurs when imaging fluorescent samples, as shown in Figure 2b. In this case, the sharpness of the 4 µm fluorescent beads images improves with integration time. Note that, in this set of experiments, 40,000 different beat frequencies were used to create these images, a number much higher than in any previous work [23], [24].

For a more quantitative assessment of the SNR of the images, we calculated this parameter as a function of the integration time, for both reflective and fluorescent samples (see further details in Supporting Information, Section S9). As shown in Figure 2c and d, in both cases the SNR increases with the square root of integration time – there is a slight deviation at longer integration times in the case of reflected light, but within the confidence interval. This dependency is characteristic of systems dominated by shot noise, thus confirming that FREMIC is ultimately limited by this type of noise. In addition, the current FREMIC implementation exhibits an apparent increase in noise at the edges of the images (Figure 2a). We attribute this effect to the AOD’s light transmission efficiency, which is lower for the frequencies located at the borders of the frequency bandwidth of the device (see further details in Supporting Information, Section S9). Importantly, though, it is possible to compensate for this inhomogeneity by normalizing the images relative to the signal from a blank reflective target – a plain mirror in current experiments.

### 3.2 Spatial resolution

**Figure 3:**
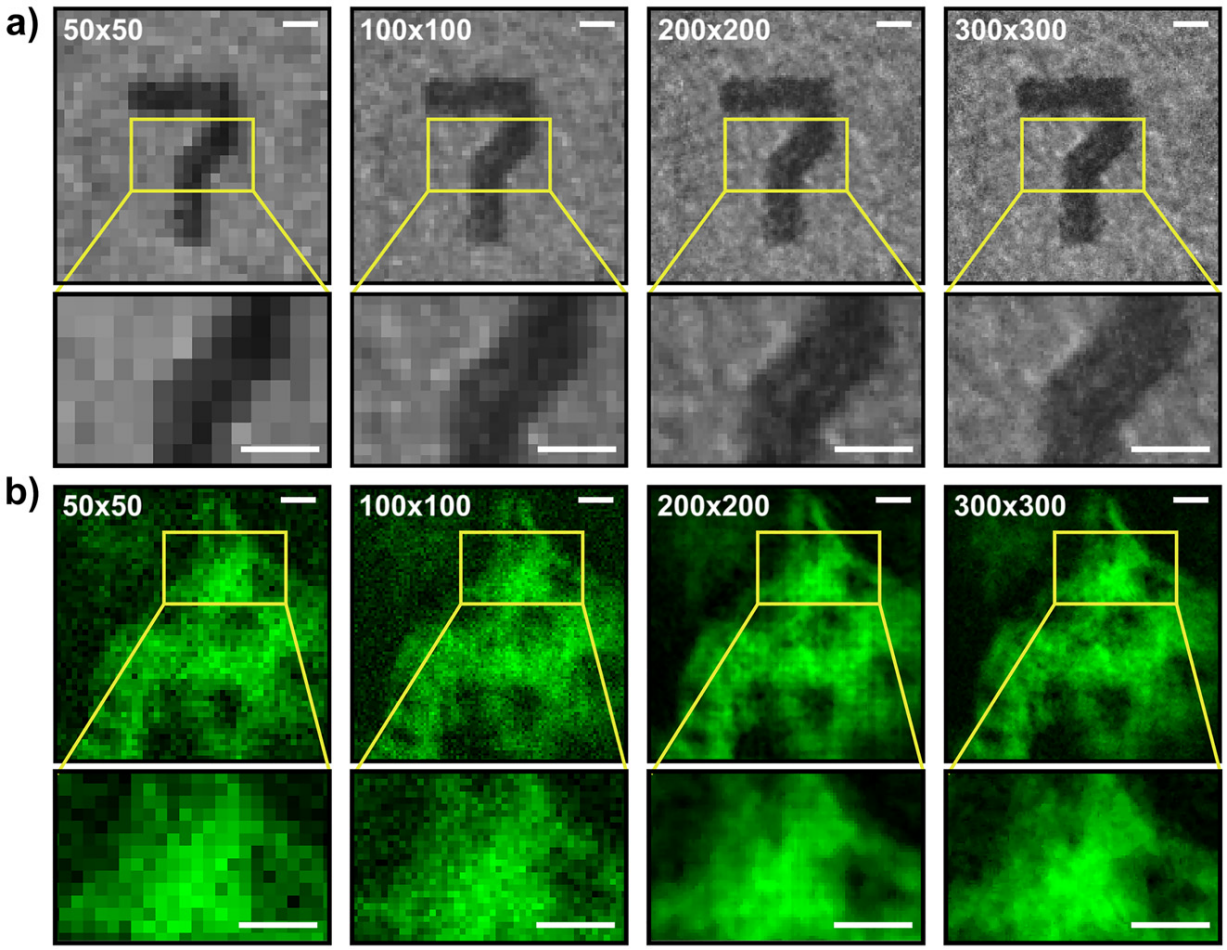
Spatial resolution of FREMIC. (a, b) Images acquired with 50 × 50, 100 × 100, 200 × 200 and 300 × 300 pixels of a reflective USAF target and a fluorescent mouse kidney section labelled with Alexa Fluor 488, showed in (a) and (b), respectively. Integration time *T* = 12 × *δt*. Scale bar 10 µm. Insets show a zoom-in of the corresponding panels. Scale bar 5 µm. Images have been normalized to visualize the same average value.

FREMIC not only allows for high imaging rates, but it is also possible to choose the number of pixels that define the reconstructed image. Indeed, the number of lines generated with the AODs determines the density of flickering points illuminating the sample, which in turn sets the spatial sampling rate and resolution. To assess how the number of flickering spots affects spatial resolution, we reconstructed images featuring a FOV of (70.3 ± 0.8) μm, at 50 × 50, 100 × 100, 200 × 200, and 300 × 300 pixels. In particular, we imaged a part of a USAF target in reflection mode (Figure 3a) and a fixed mouse kidney section in fluorescence (Figure 3b). All images were acquired using an exposure time of 12 × *δt* – note, though, that the absolute integration time differs for each pixel number, as dictated by Eq. (6). Notably, increasing the number of pixels allows for resolving finer and sharper details, as one can observe in the insets. It is also worth mentioning that the large number of frequencies simultaneously sent to the sample (up to 90,000 in current experiments) would not be possible to obtain with a single or a couple of AODs as used in previous encoding systems [23], [24]. This is due to the unique architecture of FREMIC, in which *N* driving frequencies in each AOD allow generating *N* ^2^ unique frequencies.

**Figure 4:**
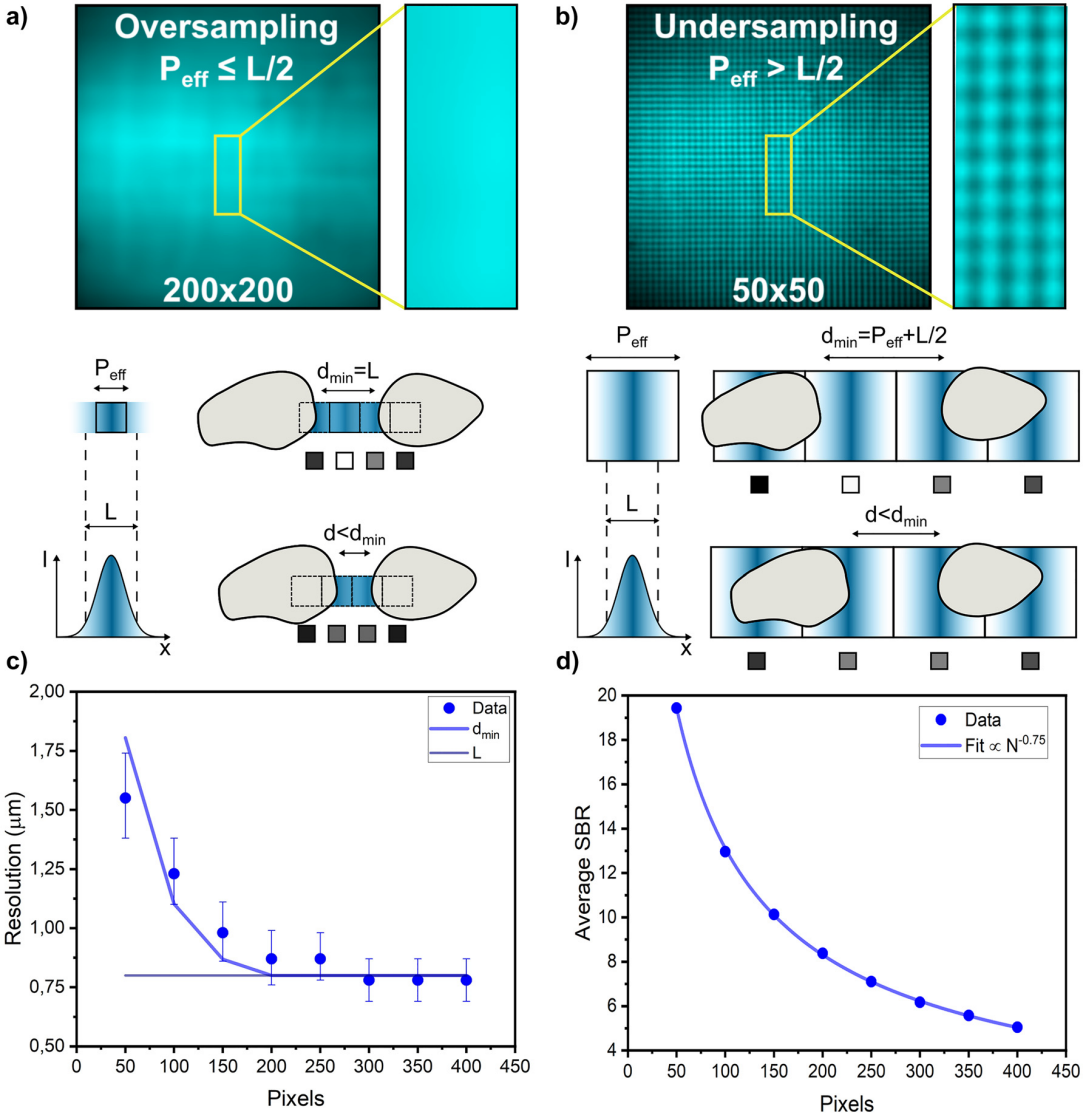
Spatial resolution and SBR characterization. (a, b) (Top) Image of a reflective plain mirror taken with a camera with 200 × 200 and 50 × 50 illuminating lines, in oversampling and undersampling condition, respectively. (Bottom) Schematic of two objects placed at relative distance *d*_min_ = *L* and *d* < *d*_min_ in oversampling condition, and at relative distance *d*_min_ = *P*_eff_ + *L*/2 and *d* < *d*_min_ in undersampling condition. The corresponding pixel size and pixel outcome are reported on the left and under each configuration, respectively. (c) Plot of *d*_min_ versus the number of pixels analyzed along one axis. The data are the minimal width of the smallest distinguishable lines of a USAF calibration target. The error bars are the line width difference between the read element and the neighbor ones. The lines are considered separated and distinguishable for a contrast value of 10 %, measured from a transverse intensity profile. In blue, *d*_min_ = *P*_eff_ + *L*/2 in undersampling and *d*_min_ = *L* in oversampling, respectively. In purple, the stripe width is plotted as a constant value; (d) plot of SBR calculated versus the number of pixels analyzed along one axis. The data and the error bars are, respectively, the mean values and the standard deviation. The data are fitted with *N* ^−0.75^.

The specific configuration of FREMIC requires a more in-depth analysis of the relationship between spatial resolution and sampling. The latter is given by the number of flickering spots, which is also the number of pixels of the reconstructed image. For a fixed FOV, we can define an effective pixel size along one axis, *P*_eff_, given by:

(8) $$P_{\text{eff}}^{i}=\frac{FOV_{i}}{N_{i}}$$

where *N* is the number of illumination lines along one axis, and the subindex *i* refers to the *x* or *y* direction. Depending on the size of *P*_eff_ relative to the diameter of the illumination stripe *L* (defined as twice the beam waist of the Gaussian light-intensity distribution), we can distinguish two different scenarios, as shown in Figure 4a and b. When *P*_eff_ ≤ *L*/2, we fulfil the Nyquist sampling condition in FREMIC, and all the object parts are illuminated. At this condition (oversampling), the spatial resolution *d*_min_ is correctly estimable and equal to *L* when the system is diffraction-limited. The second condition occurs when *P*_eff_ > *L*/2. In this case, not all object points are illuminated, resulting in undersampled images. Because only one fraction of the effective pixel is illuminated, only objects larger than *P*_eff_ can be captured. Additionally, to guarantee that two objects are distinguishable, their separation needs to be larger than $P_{\text{eff}}+\frac{L}{2}$ (Figure 4b). In such a way, independent of the absolute position of the two objects with respect to the light stripes, they would always appear separated in the reconstructed image. Note that, given the particular grid-like illumination of FREMIC where only the intersecting line strips result in flickering spots, at undersampling conditions parts of the sample can be unnecessarily exposed. Despite these constraints, undersampling could be of interest when characterizing rapidly evolving processes [31].

For a more quantitative analysis of *d*_min_ in FREMIC, we captured images of a USAF calibration target at different pixel configurations. As before, we always kept the same FOV. A plot of the minimum distinguishable feature width as a function of the number of image pixels along one axis (*N*) is presented in Figure 4c. The value of *L* in current experiments was (0.82 ± 0.01) µm (see Supporting Information, Section S3, Figure S2a), in agreement with the expected diffraction-limited size for the objective and illumination used. Interestingly, the dependency of the *d*_min_ of FREMIC with sampling (number of pixels) follows the same trend as previously discussed. Up to 170 pixels along one axis – the expected Nyquist sampling rate – undersampling occurs, and resolution scales with the number of pixels. Above 170 pixels, the resolution remains approximately constant, with a value of 780 nm. This value is in agreement with the illumination stripe diameter, confirming that FREMIC can be correctly used to reconstruct images at diffraction-limited resolution.

### 3.3 Signal to background

The control of the number of flickering spots in FREMIC does not only affect the resolution, but also the amount of light sent to each position in the sample – we kept the power of the laser source constant in our experiments. To properly characterize this effect, we first computed the signal-to-background ratio (SBR) of the reconstructed images as a function of the number of pixels along one axis. Note that we considered square images, featuring the same number of pixels in each direction. We define the Signal as the energy carried by the encoded pixels, which was calculated by multiplying the measured single-pixel intensity by the integration time. For a constant incident laser power, the single-pixel intensity decreases with the number of pixels along one axis, *N*, as *N* ^0.75^ (see Supporting Information, Section S4). Instead, the time necessary to resolve the spectral components increases quadratically with *N*, as described by Eq. (6). Therefore, the Signal captured scales as *N* ^0.25^ (Figure S2c). Regarding the Background, we define it as detector noise, that is, the measured signal obtained without illumination (laser off). Such detector noise follows a Gaussian distribution, whose power spectrum is a half-Gaussian distribution (see Supporting Information, Section S5). The average intensity of this spectrum scales with the square root of the integration time, and therefore, with *N* (see Eq. (6)). This temporal dependency is also observed in the detector current of cameras [32].

Following the definitions of signal and background and the corresponding relationship with the number of pixels along one axis, the SBR scales as ∼*N* ^−0.75^, as shown in Figure 4d. Therefore, increasing the number of pixels in our images comes at the cost of a slight decrease in SBR. For instance, a 50 × 50 image has an SBR of about 20, whereas a 300 × 300 image, with 36 times more pixels, has an SBR of 6, less than 4 times smaller. Such a trend, which is also observed for other light-sensitive metrics such as contrast (see Supporting information, Section S6), combined with the possibility of compensating for the loss of SBR by increasing the incident laser power, render FREMIC suitable for capturing images with tens of thousands of pixels.

### 3.4 Imaging of dynamic samples

**Figure 5:**
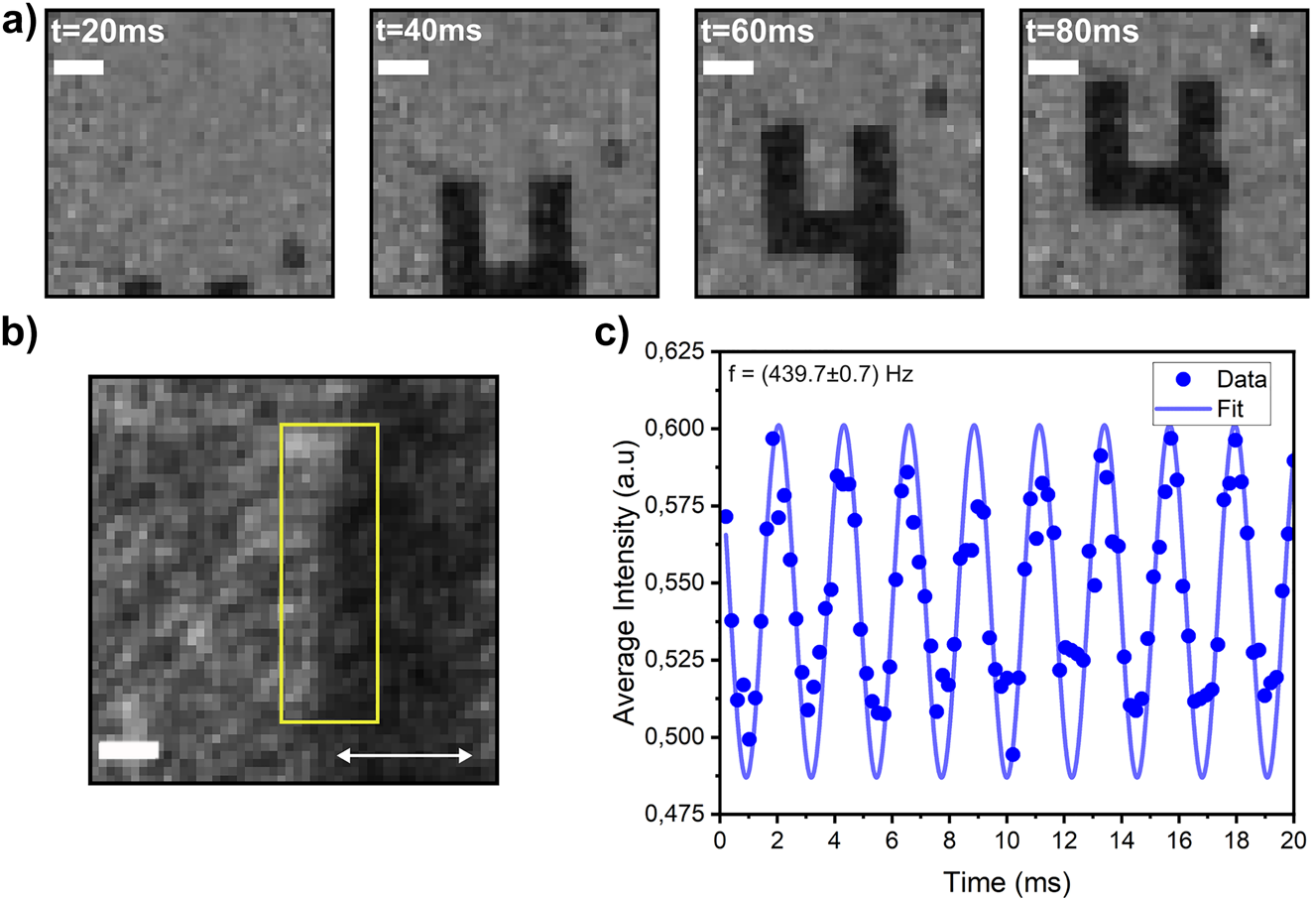
Imaging dynamic samples. (a) Cropped images of a moving USAF target, collected with an acquisition rate of 1.2 kHz. Scale bar = 10 μm. (b) 50 × 50 image of a black dot on a vibrating diapason at 440 Hz, collected with an acquisition rate of 5 kHz. Only the left edge of the dot is visible in the FOV. In Video S2, the dot oscillated along the white arrow. In the yellow area, we calculated the oscillation signal. Scale bar = 10 μm. (c) Data extrapolated from Video S2. The plotted data are the mean grey values evolving in time, extracted by the yellow area in (b). The data are fitted with a sinusoidal function with an oscillation frequency of (439.7 ± 0.7) Hz.

A distinct feature of FREMIC compared to other microscopy approaches is the possibility to select the temporal resolution of the images in a post-processing step. Thus, we can continuously record the signal of an object and then, a posteriori, select the integration time and imaging rate. This is in contrast with traditional methods in which the user needs to select the camera exposure time or scanning parameters before launching an image acquisition, which can prevent characterizing rare dynamics or non-periodic events. To prove the fast-imaging capabilities of FREMIC, we collected videos of moving samples at 50 × 50 pixels. As a first example, Video S1 shows the translation motion of a USAF target imaged with an acquisition rate of 1.2 KHz. Four frames of the video are shown in Figure 5a. Even with a low pixel density (undersampling condition) the images are well reconstructed, and the number 4 is clearly visible in each frame. As a second example, we captured a 50 × 50 pixel video of a Wittner diapason (440 Hz) at an acquisition rate of ∼5 kHz. The vibrations of the diapason were captured by marking a black dot on the instrument to partially block the reflections from its slightly polished upper surface (Video S2 and Figure 5b). A plot of the oscillation of the dot over time is shown in Figure 5c. The data exhibits a sinusoidal behavior, with an oscillation frequency of (439.7 ± 0.7) Hz, in perfect agreement with the frequency at which the diapason is designed to operate.

## 4 Conclusions

Frequency-encoded microscopy (FREMIC) allows for the recording of fast full-field scan-less images by combining acousto-optically encoded illumination with a single-pixel camera. The technique offers a customized selection of the number of pixels and the possibility of selecting the temporal resolution in a post-processing step. Compared to point or line scanning systems, FREMIC can offer improved SNR and an effective longer pixel dwell time. As our experiments demonstrate, unblurred images at sub-micrometric resolution can be captured featuring up to 400 × 400 pixels, that is more than 160,000 simultaneous frequencies, by driving two AODs with only 400 frequencies each. The maximum temporal resolution reported corresponds to 200 μs or 5000 fps (for 50 × 50 pixels), but this value is only limited by the number of pixels selected and the bandwidth of the employed AODs.

The use of commercially available AODs with larger bandwidths (up to GHz) should help to boost the acquisition rate of FREMIC by about two orders of magnitude and achieve hundreds of kHz imaging rates. The high speed of FREMIC, coupled with its relative ease of implementation and low cost, can help to expand the portfolio of optical applications to fields where fast acquisition is necessary, such as optical inspection, fluid dynamics, or plasma physics. In addition, the possibility to use single-pixel detectors sensitive to wavelengths outside the visible range can pave the way for the development of ultrafast imaging systems in the infrared and ultraviolet spectra.

## 5 Methods

### 5.1 Optical setup

A detailed schematic of the optical setup is reported in Figure 1a). The light source is a linearly polarized 488 nm continuous wave laser (Genesis MX488-1000 STM, Coherent) with an output power of 500 mW. The laser spot is expanded by a factor of 5× using a telescope (LA1951, *F* = 25.4 mm, and LA1433-A, *F* = 150 mm, Thorlabs). The laser beam is then divided by a non-polarizing beam splitter cube (BS031, Thorlabs) into two arms of an interferometer. In one of the two arms, a half-wave plate (WPH10M-488, Thorlabs), mounted on a rotating stage (RSP1X15, Thorlabs), is positioned in front of the AOD to match the required input polarization of the device. In both the orthogonal optical paths, the light is first diffracted by an AOD (ATD-7010CD2, IntraAction) and then collected by a cylindrical lens (LK1069RM-A, *F* = 200 mm, Thorlabs), mounted in a rotation cage (CRM1L/M, Thorlabs). In one optical path, the laser polarization is rotated of 90° by sending the light through another half-wave plate (WPH10M-488, Thorlabs) to match the polarization directions of the output diffracted light from the two AODs at the end of the interferometer. The two AODs are mounted on custom-made cages for fine alignment and positioning. In one of the two interferometer arms, two corner mirrors are positioned on a linear stage (M-UMR8.25, Newport) for fine adjustment of the path length. The two light beams are recombined in a non-polarizing beam splitter cube (CCM1-BS013/M, Thorlabs) and successively relayed 1:1 to the second lens of the telescope (LA1708-A, *F* = 200 mm, Thorlabs). Afterward, the light is guided through a periscope into a scan lens (LA1484-A, *F* = 300 mm, Thorlabs), and optically coupled with a tube lens (LA1708-A, *F* = 200 mm, Thorlabs). Here the beam is reflected by a beam splitter plate (BSW10R, Thorlabs) or by a dichroic mirror (FITC Filter Cube Set – Nikon) into a 40× objective (Nikon CFI Plan Fluor, NA 0.75, Nikon) and finally directed to the sample. In detail, the samples used in the experiments are two different USAF calibration targets (R3L3S1PR positive reflective, Thorlabs, and RTA39D22 positive reflective PhotomaskPortal, respectively), 4 µm fluorescent beads (TetraSpeck T14792), a fixed mouse kidney section labelled with Alexa Fluor 488 (Invitrogen F24630 – Thermo Fisher Scientific) and a square Wittner diapason. The reflected, or fluorescent, light is retraced back into a detection telescope (LA1708-A, *F* = 200 mm, and LA1908-A, *F* = 500 mm, Thorlabs) and collected by a 10× objective (Nikon S Fluor, NA 0.5) onto a silicon avalanche photodetector (APD430A2/M, Thorlabs, variable gain, 400 MHz bandwidth). The corresponding voltage signal is sampled by a fast oscilloscope board (CobraMax CS23G8, GaGe Applied Technologies), exactly synchronized with a digital delay generator (DG645, Stanford Research Systems). For images in Figure 4a and b as-CMOS camera (PCO.edge 4.2 bi, Excelitas Technologies Corp) has been used instead of the APD.

### 5.2 AOD driving signal

The driving signals for the two AODs are generated by a high-speed four-channel digital-to-analog conversion board (PXDAC4800, Signatec) which works as an arbitrary waveform generator at a maximum rate of 1.2 GHz. For each AOD, the signal generated is the sum of *N* sine waves with chosen random phases, such that the total amplitude modulation is minimized. Before entering the AODs the signals are also properly amplified by two deflector drivers (DE-704M, IntraAction) with a bandwidth centered at 70 MHz. The results described in this work were obtained by setting an effective bandwidth of Δ*f*_*y*_ = 12 MHz for one AOD, while for the other, we used:

(10) $$\Delta f_{x}=\Delta f_{y}\left(1-\frac{1}{N}\right)$$

That shows how the two bandwidths slightly differ, depending on the number of beams generated. Further details about the derivation of Eq. (10) are reported in Supporting Information (Section S1). There, it is shown how Eq. (10) represents a strict constraint to obtain the minimum integration time introduced in Eq. (6).

## Supplementary Material

Supplementary Material Details

## References

[1] Suzuki C. T. N., Gomes J. F., Falcao A. X., Shimizu S. H., Papa J. P. (2013). Automated diagnosis of human intestinal parasites using optical microscopy images. *2013 IEEE 10th International Symposium on Biomedical Imaging*.

[2] Adur J. (2014). Colon adenocarcinoma diagnosis in human samples by multicontrast nonlinear optical microscopy of hematoxylin and eosin stained histological sections. *J. Cancer Ther.*.

[3] Sancataldo G., Silvestri L., Letizia A., Mascaro A., Sacconi L., Pavone F. S. (2019). Advanced fluorescence microscopy for in vivo imaging of neuronal activity. *Optica*.

[4] Alexandropoulos C., Duocastella M. (2023). Video-rate quantitative phase imaging with dynamic acousto-optic defocusing. *Opt Laser. Eng.*.

[5] Sioma A. (2023). Vision system in product quality control systems. *Appl. Sci.*.

[6] Ji N., Freeman J., Smith S. L. (2016). Technologies for imaging neural activity in large volumes. *Nat. Neurosci*..

[7] Brandenburg B., Zhuang X. (2007). Virus trafficking – learning from single-virus tracking. *Nat. Rev. Microbiol.*.

[8] Blasi T. (2016). Label-free cell cycle analysis for high-throughput imaging flow cytometry. *Nat. Commun.*.

[9] Vilar N. (2022). Optical system for the measurement of the surface topography of additively manufactured parts. *Meas. Sci. Technol*..

[10] Kodama R. (2001). Fast heating of ultrahigh-density plasma as a step towards laser fusion ignition. *Nature*.

[11] Edgar M. P., Gibson G. M., Padgett M. J. (2019). Principles and prospects for single-pixel imaging. *Nat. Photonics*.

[12] Nwaneshiudu A., Kuschal C., Sakamoto F. H., Rox Anderson R., Schwarzenberger K., Young R. C. (2012). Introduction to confocal microscopy. *J. Invest. Dermatol.*.

[13] Helmchen F., Denk W. (2005). Deep tissue two-photon microscopy. *Nat. Methods*.

[14] So P. T. C., Dong C. Y., Masters B. R., Berland K. M. (2000). Two-photon excitation fluorescence microscopy. *Annu. Rev. Biomed. Eng*..

[15] Hahamovich E., Monin S., Hazan Y., Rosenthal A. (2021). Single pixel imaging at megahertz switching rates via cyclic hadamard masks. *Nat. Commun.*.

[16] Chen B., Guo Y., Sun B., Jiao J., Wang Y., Jiang W. (2021). Single-pixel camera based on a spinning mask. *Opt. Lett.*.

[17] Wu J., Hu L., Wang J. (2021). Fast tracking and imaging of a moving object with single-pixel imaging. *Opt. Express*.

[18] Goda K., Tsia K. K., Jalali B. (2009). Serial time-encoded amplified imaging for real-time observation of fast dynamic phenomena. *Nature*.

[19] Tsia K. K., Goda K., Capewell D., Jalali B. (2010). Performance of serial time-encoded amplified microscope. *Opt. Express*.

[20] Gao L., Liang J., Li C., Wang L. V. (2014). Single-shot compressed ultrafast photography at one hundred billion frames per second. *Nature*.

[21] Mikami H., Gao L., Goda K. (2016). Ultrafast optical imaging technology: principles and applications of emerging methods. *Nanophotonics*.

[22] Tsyboulski D., Orlova N., Saggau P. (2017). Amplitude modulation of femtosecond laser pulses in the megahertz range for frequency-multiplexed two-photon imaging. *Opt. Express*.

[23] Mikami H. (2018). Ultrafast confocal fluorescence microscopy beyond the fluorescence lifetime limit. *Optica*.

[24] Diebold E. D., Buckley B. W., Gossett D. R., Jalali B. (2013). Digitally synthesized beat frequency multiplexing for sub-millisecond fluorescence microscopy. *Nat. Photonics*.

[25] Berezin M. Y., Achilefu S. (2010). Fluorescence lifetime measurements and biological imaging. *Chem. Rev*..

[26] Mizuno T. (2021). Full-field fluorescence lifetime dual-comb microscopy using spectral mapping and frequency multiplexing of dual-comb optical beats. *Sci. Adv*..

[27] Reddy G. D., Saggau P. (2005). Fast three-dimensional laser scanning scheme using acousto-optic deflectors. *J. Biomed. Opt.*.

[28] Duocastella M., Surdo S., Zunino A., Diaspro A., Saggau P. (2021). Acousto-optic systems for advanced microscopy. *J. Phys. Photon.*.

[29] Sarder P., Maji D., Achilefu S. (2015). Molecular probes for fluorescence lifetime imaging. *Bioconjugate Chem.*.

[30] Zunino A. (2021). Multiplane encoded light-sheet microscopy for enhanced 3D imaging. *ACS Photonics*.

[31] Chan A. C. S., Tsia K. K., Lam E. Y. (2016). Subsampled scanning holographic imaging (SuSHI) for fast, non-adaptive recording of three-dimensional objects. *Optica*.

[32] Reibel Y., Jung M., Bouhifd M., Cunin B., Draman C. (2003). CCD or CMOS camera noise characterisation. *EPJ Appl. Phys.*.

