# Supplementary material for: Scan-less microscopy based on acousto-optic encoded illumination
Source: Nanophotonics. 2024 Jan 2;13(1):63–73. doi: 10.1515/nanoph-2023-0616 (PMC10790963; doi:10.1515/nanoph-2023-0616)
Supplement: Supplementary file 1 — Supplementary Material Details [file j_nanoph-2023-0616_suppl_003.docx]

**Supporting information:**

**Scan-less microscopy based on acousto-optic encoded illumination**

Andrea Marchese^1,§^, Pietro Ricci^1,§^, Peter Saggau^2^, Martí Duocastella^1,*^

^1^Department of Applied Physics, Universitat de Barcelona, Barcelona, Spain

^2^Department of Neuroscience, Baylor College of Medicine, Houston, TX, USA

^§^Co-first authors contributed equally

*Corresponding author e-mail: marti.duocastella@ub.edu

Contents

[Section S1. DERIVATION OF THE MINIMUM ACQUISITION TIME 3](#_Toc149488426)

[Section S2. SNR COMPARISION WITH POINT-BY-POINT SCANNING SYSTEM 5](#_Toc149488427)

[Section S3. PIXEL SIZE, FIELD-OF-VIEW, AND STRIPE WIDTH EVALUATION 6](#_Toc149488428)

[Section S4. SIGNAL CONSIDERATIONS AND EVALUATION 7](#_Toc149488429)

[Section S5. BACKGROUND CONSIDERATIONS AND EVALUATION 8](#_Toc149488430)

[Section S6. CONTRAST EVALUATION 9](#_Toc149488431)

[Section S7. AOD’s TRANSMISSION 10](#_Toc149488432)

[Section S8. WAVEFORM ANALYSIS 10](#_Toc149488433)

[Section S9. SNR EVALUATION 12](#_Toc149488434)

[REFERENCES 12](#_Toc149488435)

# Section S1. DERIVATION OF THE MINIMUM ACQUISITION TIME

The presented frequency-encoded microscopy is based on the interference of orthogonal light stripes, frequency-modulated by acoustic waves traveling in an AOD. In particular, evenly spaced frequencies must be used to generate light stripes that are evenly spaced. However, it is not guaranteed that two sets of these light stripes will produce a pattern of crossing points with unique beat frequencies. In this section, we illustrated how choosing the AOD’s acoustic frequencies to have unique beat frequencies and the corresponding acquisition rate.

Let’s consider that the two AODs are crossed by N acoustic waves simultaneously with frequencies f_i_ and f_j_, where i, j = 1…N are indices of the first and the second AOD, respectively (along x and y). The N frequencies generated by AODs are evenly distributed over the bandwidth Δf_x_ and Δf_y_ respectively, such that:

$f_{i}=f_{Cx}-\frac{{\Delta f}_{x}}{2}+(i-1)\frac{{\Delta f}_{x}}{N-1}$ (s1)

where f_Cx_ is the bandwidth central frequency. An analogous relation is valid for the other AOD. Then, the laser light of frequency 𝜈_L_ enters the AOD and is frequency-shifted by the Doppler effect as:

$F_{i}=\nu_{L}+f_{i}$ (s2)

These are the frequencies encoding each diffracted beam - and so the light stripes - exiting the AODs and composing the crossing pattern. As a consequence, the light intensity at the crossing point regions has an interference-modulated amplitude, with a beat frequency of:

$F_{ij}=F_{i}-F_{j}$ (s3)

Let us define now the spectral distance between beat frequencies:

$\Delta F=\left| F_{ij}-F_{i'j'} \right|$ (s4)

Using Eq.s1-s3, we can rewrite eq.s4 as:

$\Delta F=\frac{{\Delta f}_{y}}{N-1}\left| \left( j´-j \right)-\Delta_{R}\left( i´-i \right) \right|$ (s5)

Where $\Delta_{R}={{\Delta f}_{x}}/{{\Delta f}_{y}}$ is the bandwidth ratio, and ${\Delta f}_{y}\geq{\Delta f}_{x}\geq0$ without loss of generality. Since we want unambiguous decoding for every position in the grid pattern it is necessary to ensure that all beat frequencies are unique, which means ΔF must never be zero. Then, we define 𝛿F as the distance between the closest beat frequencies:

$\delta F={min}_{i,j,i',j'}\left\{ \Delta F \right\}$ (s6)

It is required that 𝛿F≠0 to avoid ambiguity. This is satisfied for:

$\frac{{\Delta f}_{y}}{N-1}\left| \left( j´-j \right)-\Delta_{R}\left( i´-i \right) \right|$≠0 (s7)

For every (i,j)≠(i’,j’). It also means:

$\frac{{\Delta f}_{x}}{{\Delta f}_{y}} \neq\frac{\left( j´-j \right)}{\left( i´-i \right)}$ (s8)

Since (j’-j) and (i’-i) are all integers ranging from 0 to N-1, Eq.s8 can be also rewritten as:

$\frac{{\Delta f}_{x}}{{\Delta f}_{y}} \neq\frac{l}{k}$ (s9)

Where $l$ and $k$ are such that $0\leq l\leq k<N$. It is noteworthy that the so-defined rational numbers $l/k$ describe the Farey sequence of order N-1 (FS_N-1_)^1^. Hence, recalling the definition of Δ_R_, we must have:

$\Delta_{R}\neq{FS}_{N-1}$ (s10)

At this point, it is important to highlight that 𝛿F determines the maximum speed of our imaging system (and the minimum exposure time 𝛿t necessary). On the other part, the frequency sampling df of a Fourier transform - and so the ability to resolve close spectral components - is inversely proportional to the integration time T. Summarizing, we have:

$\frac{1}{T}=df\leq\delta_{F}$ (s10)

Then, to acquire signals for a time window as short as possible, we have to find the maximum 𝛿F. Let us study this in the neighborhood of Δ_R_, namely in a region $\Delta_{R}=l/k\pm\varepsilon$ where ε is small. From Eq.s5 one obtains:

$\delta F( l/k \pm\varepsilon) ={min}_{i,j,i',j'}\left\{ \frac{{\Delta f}_{y}}{N-1}\left| \left( j´-j \right)-\frac{l}{k}\left( i´-i \right) \pm\varepsilon\left( i´-i \right) \right| \right\}$ (s11)

For ε→0, Eq.s11 must vanish, as discussed above in Eq.s7-s9. The indices that satisfy this requirement are $\left( i´-i \right)=\pm k$ and $\left( j´-j \right)=\pm l$. For which Eq.s11 becomes:

$\delta F( l/k \pm\varepsilon) ={min}_{i,j,i',j'}\left\{ \frac{{\Delta f}_{y}}{N-1}\cdot\left| \pm\varepsilon k \right| \right\}$ (s12)

Since:

$\varepsilon=\pm\Delta_{R}\mp l/k$ (s13)

For which Eq.s12 becomes:

$\delta F(\Delta_{R})=\frac{{\Delta f}_{y}}{N-1}\left| l-k\Delta_{R} \right|$ (s14)

Thus, 𝛿F(Δ_R_) is a piecewise linear function, whose slope and intersects depend on the values of the Farey sequence they are close to. Therefore, we may expect a linearly increasing trend for values slightly larger than $l/k$, which starts to decrease linearly when Δ_R_ approaches the next Farey sequence point $q/h$ (where ${0\leq l}/{k<q/h}\leq1$). Then, there must be a local maximum between two consecutive Farey points $l/k$ and $q/h$. The two linear trends can be described by:

$\frac{{\Delta f}_{y}}{N-1}\left| l-k\Delta_{R} \right|$ for $\Delta_{R}\gtrsim\frac{l}{k}$ (s15a)

$\frac{{\Delta f}_{y}}{N-1}\left| q-h\Delta_{R} \right|$ for $\Delta_{R}\lesssim\frac{q}{h}$ (s15b)

They match at:

$\Delta_{R}=\frac{(l+q)}{(k+h)}$ (s16)

The point $\left( l+q \right)/\left( k+h \right)$ is known in the literature as mediant of Farey points $l/k$ and $q/h$. The last requirement is to illuminate the sample as homogeneously as possible, which means choosing Δ_R_ close to 1. The mediant of interest is the one obtained with the two last Farey points (N-2)/(N-1) and 1/1, namely:

$\Delta_{R}=\frac{N-2+1}{N-1+1}=\frac{N-1}{N}$ (s17)

Knowing this, all the images and videos presented in this work were collected by setting:

${\Delta f}_{x}={\Delta f}_{y}(1-\frac{1}{N})$ (s18)

Finally, substituting Eq.s17 in Eq.s14 we find:

$\delta F=\frac{{\Delta f}_{y}}{N-1}\left| 1-\Delta_{R} \right|=\frac{{\Delta f}_{y}}{N(N-1)}=\frac{1}{\delta t}$ (s19)

This means the minimum integration time is dependent on the number of acoustic frequencies sent to the AODs.

We verified experimentally the validity of Eq.s19 by shining a pattern on a reflective USAF target and measuring the spectrum. We generated a 50x50 pattern with ${\Delta f}_{y}=12$MHz and ${\Delta f}_{x}$ as given by Eq.s18 (Fig.S1a). The spectrum shown in Fig.S1b displays a multitude of peaks. The expected frequency positions of the most prominent peaks match with all the 2500 expected F_i,j_ (Fig.S1c). Furthermore, the distance between the closest peaks corresponds to the 𝛿F described by Eq.s19, showing a good agreement between theory and experiments.


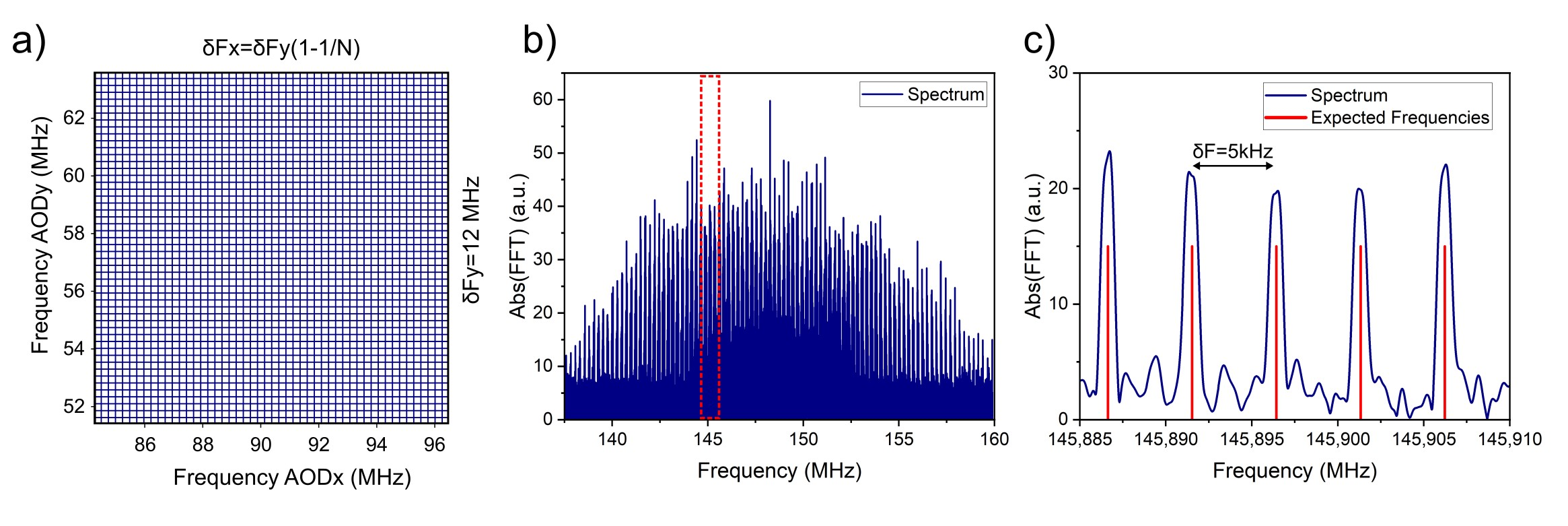


**Fig.S1:** **a)** Simulation of a 50x50 pattern considering ${\Delta f}_{x}$ and ${\Delta f}_{y}$ as indicated on the side of the plot axes. The horizontal (vertical) lines represent the acoustic frequencies of the AOD used in the first (second) arm of the Mach Zender interferometer. **b)** Spectrum of an experimental time trace of duration 24x𝛿t. **c)** Zoom-in of the red dashed area shown in b), and expected frequencies positions (red lines).

# Section S2. SNR COMPARISION WITH POINT-BY-POINT SCANNING SYSTEM

To compare the signal to noise ratio (SNR) between a scan-less system such as FREMIC and a point-by-point scanning system, we can first calculate the differences in the retrieved signal and noise. For simplicity, we consider the total imaging time T and the radiative power I in a single spot to be equal in the two systems. This also implies that the time spent to collect the light from a single pixel in a scanning system is T/(N×N). Considering the typical sources of noise to be shot noise (sn) and detector noise (dn), originating from the stochastic nature of photon collection and other external signal-independent causes (electric, environmental, etc.), respectively, we can write^2^:

$Signal=RI$ $Noise=\sqrt{(2e(i_{sn}+i_{dn})\Delta\nu}$ (s20)

where $e$ is the electron charge, $i_{sn}$ and $i_{dn}$ are the shot noise and the detector noise currents, respectively, and $\Delta\nu$ is the inverse of the single-pixel integration time –$N^{2}/T$ for a scanning system, and $1/T$ for the scan-less case. Let us call R the detector responsivity, we have that $i_{sn}=RI$ and $i_{sn}=N^{2}\cdot RI$, for a scanning and scan-less system, respectively. The latter is due to the total amount of radiating points contributing simultaneously to the signal. Thus, the ratio between the SNR calculated for the two approaches can be finally written as:

$\frac{{SNR}_{scan-less}}{{SNR}_{scanning}}=\frac{\sqrt{(2e(RI+i_{dn})\frac{N^{2}}{T}}}{\sqrt{(2e(N^{2}\cdot RI+i_{dn})\frac{1}{T}}}$ (s21)

# Section S3. PIXEL SIZE, FIELD-OF-VIEW, AND STRIPE WIDTH EVALUATION

For a spatial characterization of our system, we measured the Pixel size (P_size_), the field-of-view (FOV) and the width of the light stripe (L). To measure P_size_ we selected as reference an object of known size, i.e. the first element of the fifth group of a USAF target (R3L3S1PR, Thorlabs), with a line width LW_e1.g5_=15.63 μm. With the configuration of the largest number of pixels between the one analyzed, i.e. the 400$\times$400 pixels, we measured the width of the reference to be (89±1) pixels. From this, we obtained the pixel size P_size_(400 pixels) = (0.176±0.002) μm. Then, we concluded that the field covered by the illumination grid is FOV = P_size_xN = (70.3±0.8) μm. For completeness, the P_size_ for the other pixel configurations can be straightforwardly obtained by P_size_(N)=FOV/N. In the end, since the bandwidth used for the experiments was Δ_y_=12 MHz ≃Δ_x_, we obtained a conversion factor of γ = (5.85±0.07) μm/MHz.

After that, we measured the width of the light stripe projected on the sample. For this experiment, we made use of only one AOD at a time, blocking the light transmission through the other interferometer arm. In particular, we first determined the distance between two light stripes by observing the intensity of their beating frequency in the spectral domain. In detail, we gradually changed the driving frequency of one of the two diffracted stripes, bringing them closer, and observing their overlap becoming prominent. Notably, the signal arising from the overlap of two stripes is the convolution of the two single signals. Therefore, since the two stripes generated with the AODs had a transversal Gaussian intensity profile, it is legitimate to assume that also the convolution can be described with the same trend. Therefore, the sigma of the convolution is double the sigma of the single curves.

Fig.S2a shows the intensity of the peaks obtained using the two AODs consecutively, reported in blue and red, respectively. Note how, as the distance between two light stripes decreases, the peak corresponding to their frequency difference increases. The two profiles were fitted with Gaussian curves, from which we extracted the waist (1/e^2^) w_AOD1_= (140±2) kHz and w_AOD2_=(158±2) kHz. Following the discussion above and using the conversion factor γ we concluded that the widths L of the light stripes generated with the AODs and projected on the sample, were respectively L_AOD1_=(0.82±0.01) μm and L_AOD2_=(0.92±0.01) μm. We suspect that the slight discrepancy ΔL=0.1 μm between the two widths is mainly due to a small misalignment between the optics position of the two arms of the interferometer.

For the sake of comparison, we also estimated the diffraction-limited width of the light stripe in our system according to Rayleigh’s criterion. In particular, we considered the illumination objective with a nominal focal length of 5 mm for a 40x magnification (the nominal tube lens has a focal length of 200 mm). Finally, we measured the width of the light beam at the back pupil of the first objective to be D=3.8 mm. For the wavelength of 488 nm, we had:

$L_{Rayleigh}=1.22\cdot\lambda\cdot\frac{F}{D}=1.22\cdot0.488\cdot\frac{5}{3.8}=0.8 \mu m$ (s22)

This is in good accordance with the value found before.

# Section S4. SIGNAL CONSIDERATIONS AND EVALUATION

Few physical considerations concerning the AOD-based setup allow us to understand the Signal expected trend as a function of the number of pixels considered.

As a first step, let us consider the light transmitted from the AOD in each interferometer arm. The input laser power P^L^ is divided into two equal parts at the entrance of the interferometer. This amount is then split between the 0^th^ order and the other N-diffracted beams. However, the N^th^-beam output power does not depend linearly on the input power. Still, it is a general function of the amplitude and phase of the frequency component in which the radiofrequency generator output signal is divided. For this reason, we maintain a general dependency on N^𝛼^:

$\sum_{i=1}^{N} P_{i}\propto\frac{P^{0}}{N^{\alpha}}$ (s23)

Where P_i_ is the power carried by the N-th diffracted beam, and P^0^ is the power of the first order if we would set N=1. If we introduce now the average power carried by each diffracted beam:

$\bar{P}=\frac{\sum_{i=1}^{N} P_{i}}{N}$ (s24)

Using Eq.s23, we can rewrite Eq.s24 as follows:

$\bar{P}\propto\frac{P^{0}}{N\cdot N^{\alpha}}$ (s25)

Now, the signal carried by each pixel in an image is directly correlated with the energy carried in each stripe intersection at the sample plane. This energy depends on the power developed by the interference of two intersecting stripes, and on the integration time that we are considering to collect the signal.

$W_{Pixel}=P_{Pixel}\cdot T$ (s26)

The integration time necessary to resolve the Fourier spectrum is fully described by Eq.s19, which highlights a strong dependency on the number of pixels (∼N^2^). For what concerns the power carried by each pixel at the sample plane, is determined by the interference of electric fields of two crossing stripes (respectively E_AOD1_ and E_AOD2_). Since their amplitude can be obtained by the square root of the carried power, and recalling Eq.s25, we can rewrite Eq.s26 as:

$W_{Pixel} \propto\left| E_{AOD1} \right|\cdot\left| E_{AOD2} \right| \cdot\delta t \propto\sqrt{\bar{P}_{AOD1}}\sqrt{\bar{P}_{AOD2}}$ $\cdot\delta t \propto\frac{1}{N^{\frac{1}{2}}\cdot N^{\frac{\alpha}{2}}}\cdot\frac{1}{N^{\frac{1}{2}}\cdot N^{\frac{\beta}{2}}}\cdot N^{2}=\frac{N}{N^{\frac{\alpha+\beta}{2}}}$ (s27)

Where $\alpha$ and $\beta$ describe the transmission non-linearity previously mentioned, respectively for AOD_1_ and AOD_2_. In each interferometer arm, the laser power transmitted from the AOD on the N-diffracted beams was measured with a photodiode power sensor (S121C, Thorlabs). The goal of this measure was the evaluation of the transmitted power dependency on the number of stripes simultaneously diffracted, i.e. a measure of $\alpha$ and $\beta$. In particular, the sensor was positioned in a Fourier plane after both the AODs, alternating the illumination from one and the other arm. In this position, the power meter collected and integrated the contribution of all the diffracted beams at once (except the 0^th^ order):

$P_{M}=\sum_{i=1}^{N} P_{i}=N\cdot\bar{P}$ (s28)

Recalling Eq.s25, we can rewrite the measured power as:

$P_{M}=N\cdot\frac{P^{0}}{N\cdot N^{\alpha}}=\frac{P^{0}}{N^{\alpha}}$ (s29)

Fig.S2b shows the results obtained for the two devices in blue and red respectively. In particular, we plotted the data in a log-log scale to linearly fit them and extrapolate the slope $\alpha$ and $\beta$.

Even if the laser output power was perfectly spitted in the two arms of the interferometer, a slightly different efficiency of the two AOD transmissions is evident. Indeed the results were:

- $\alpha$= - (0,65±0,03)
- $\beta$= - (0,85±0,02)

At this point, we fit the signal values extrapolated from the images with the trend described in Eq.s27:

$W_{Pixel} \propto\frac{N}{N^{\frac{\alpha+\beta}{2}}}=N^{1-0.75}=N^{0.25}$ (s30)

As shown in Fig.S2c, the data were in good accordance with the model described.

# Section S5. BACKGROUND CONSIDERATIONS AND EVALUATION

Let’s consider the discrete sampling of a random background signal V(t). This sampling produces a sequence of values V_n_ that we can assume to be statistically uncorrelated, and that can be described by a Gaussian distribution with standard deviation σ. Now, the discrete Fourier transform (DFT) of a Gaussian noise function is another Gaussian noise function with width:

$\sigma_{FT}=\sigma\cdot\sqrt{\frac{n}{2}}$ (s31)

Where n is the number of samples. Then in detail, to extract the amplitude of the frequency component from the spectrum, we considered the modulus of the values, changing the original distribution to a half-normal distribution. The mean of the probability density function half-normal distributed is:

$\bar{\mu}=\frac{\sigma\cdot\sqrt{2}}{\sqrt{\pi}}$ (s32)

Where $\sigma$ is the standard deviation of the original normal distribution. Finally, we can safely conclude that the average background signal goes itself with the square root of the number of sampled points. Now let us define the number of samples as:

$n=\frac{T}{dt}$ (s33)

Where T is the total integration time and dt is the time resolution, obtained by inverting the sampling rate of the acquisition board (375 MegaSample/s). In the end, since the number of samples n scales with the integration time T, and recalling Eq.s19, we obtain the pixel dependency of the average background signal as:

$\mu_{background} \propto\sqrt{n} \propto\sqrt{T}\propto\sqrt{N^{2}}=N$ (s34)

Fig.S2d shows the average background signal obtained from the measurements, fitted with a linearly increasing function of the number of pixels. Results are in good agreement with the discussed trend.


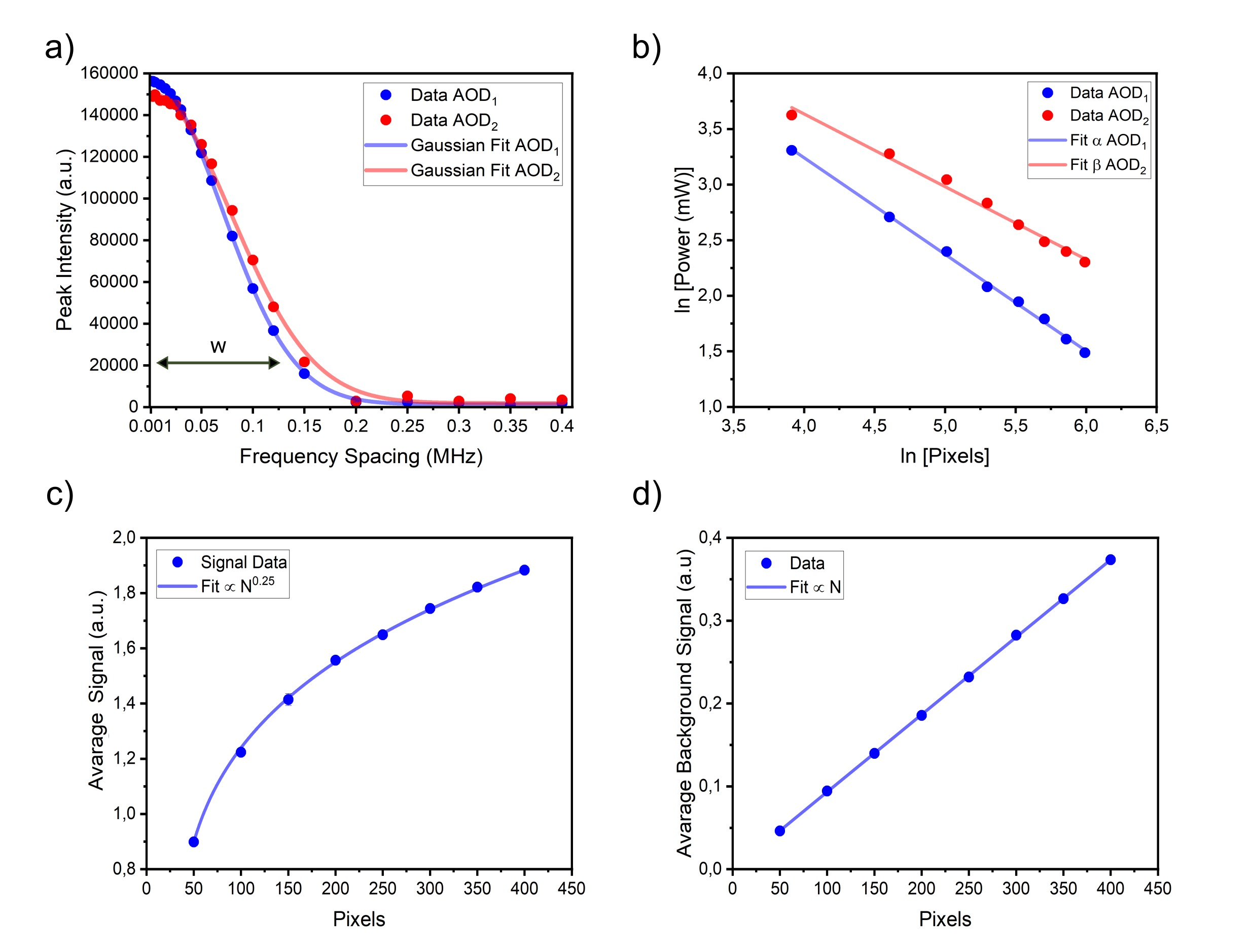


**Fig.S2: a)** Interference peak intensity between two adjacent stripes generated with AOD_1_ (in red) and alternatively with AOD_2_ (in blue), as a function of their distance (in MHz). From the spectrum, amplitudes were extracted for the frequency component relative to their interference, namely f_1_-f_2_. Data are fitted with a Gaussian profile, from which the beam waist was extracted. Acquisition time T=2 ms. Data mean values and standard deviations were obtained over 300 acquisitions. **b)** Measurement of the total AOD transmitted power on the N diffracted beams, measuring singularly for AOD_1_ (in blue) and for AOD_2_ (in red). Data are plotted in a log-log scale to be linearly fitted and to extract the non-linear transmission coefficients $\alpha$ and $\beta$. **c)** Plot of the measured signal for all the pixels configuration analysed. Data and error bars are the mean values and the standard deviation, respectively, obtained over 5 repeated measurements. Data are fitted with N^0.25^ as proposed in the model. **d)** Plot of the measured background for all the pixels configuration analysed. Data and error bars are respectively, mean values and standard deviation obtained over 5 repeated measurements. Data are fitted with a linear function of number of pixels, as proposed in the model.

# Section S6. CONTRAST EVALUATION

We evaluated the image contrast from the same dataset used to estimate the Signal-to-Background ratio. In this case, we calculated the contrast using:

$C=\frac{I_{Signal} -I_{Background}}{I_{Signal} + I_{Background}}$ (s35)

Fig.S3 shows the contrast measured for all the selected pixel configurations, from 50x50 up to 400x400. The fit function is obtained by analyzing separately the different terms discussed in following the discussed in “Signal and Background consideration and evaluation” section.

This trend confirms that the image contrast decreases by increasing the number of stripes projected on the sample. The intensity decreasing with the number of illuminated spots is a common feature of all the existing light parallelization techniques. However, this trend can be easily flattened by pre-calibration and fine regulation of the input illumination power**.**


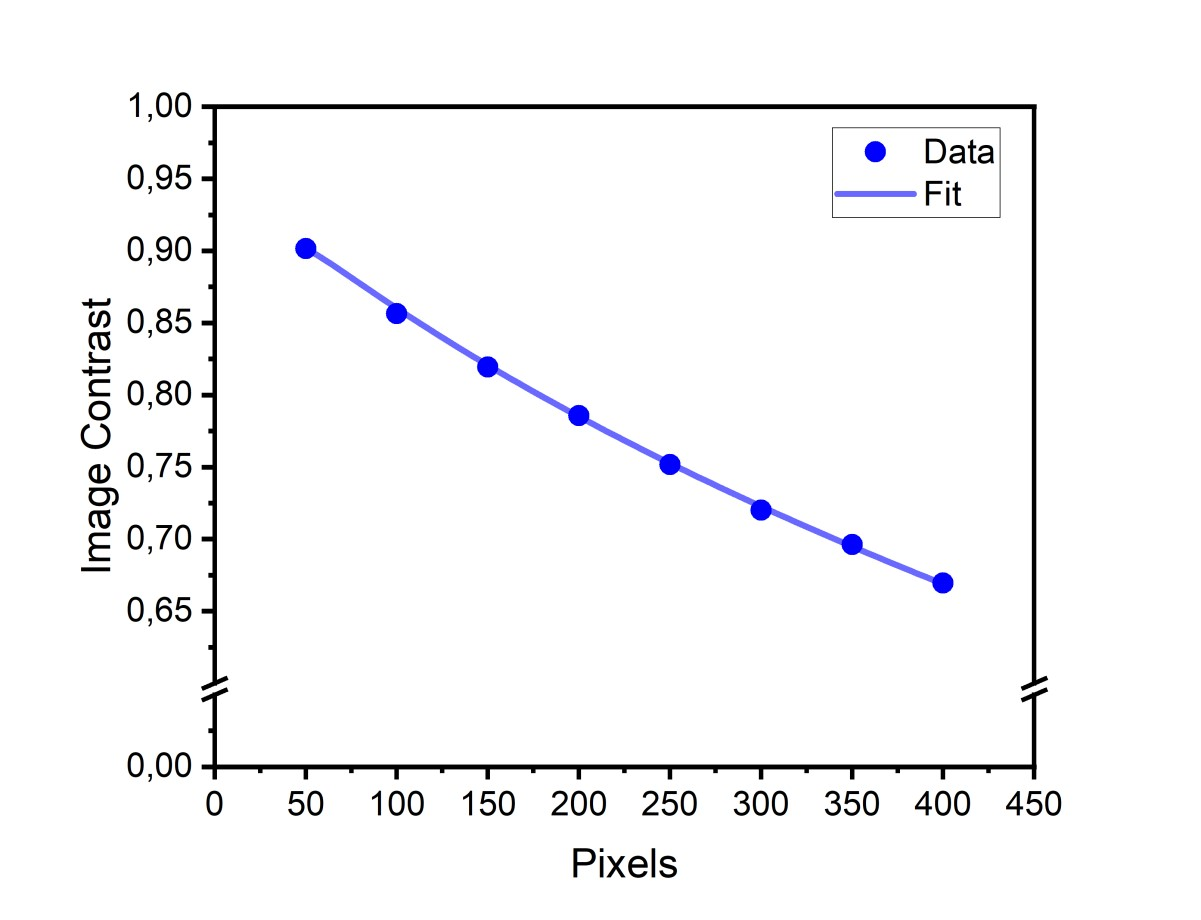


**Fig.S3:** Contrast calculated for different configurations analyzed, from 50$\times$50 up to 400$\times$400 pixels. Data and error bars are respectively mean values and standard deviation. Data are fitted as proposed from the model.

# Section S7. AOD’s TRANSMISSION

We evaluated quantitatively the AOD’s light transmission efficiency by calculating the SNR for each pixel of a 200x200 image of a blank reflective target (Fig.S4). The SNR is clearly higher at the central region, whereas it progressively decays toward the borders. Note how, probably due to the different AODs’ frequency response, this effect is more evident along one of the two directions.


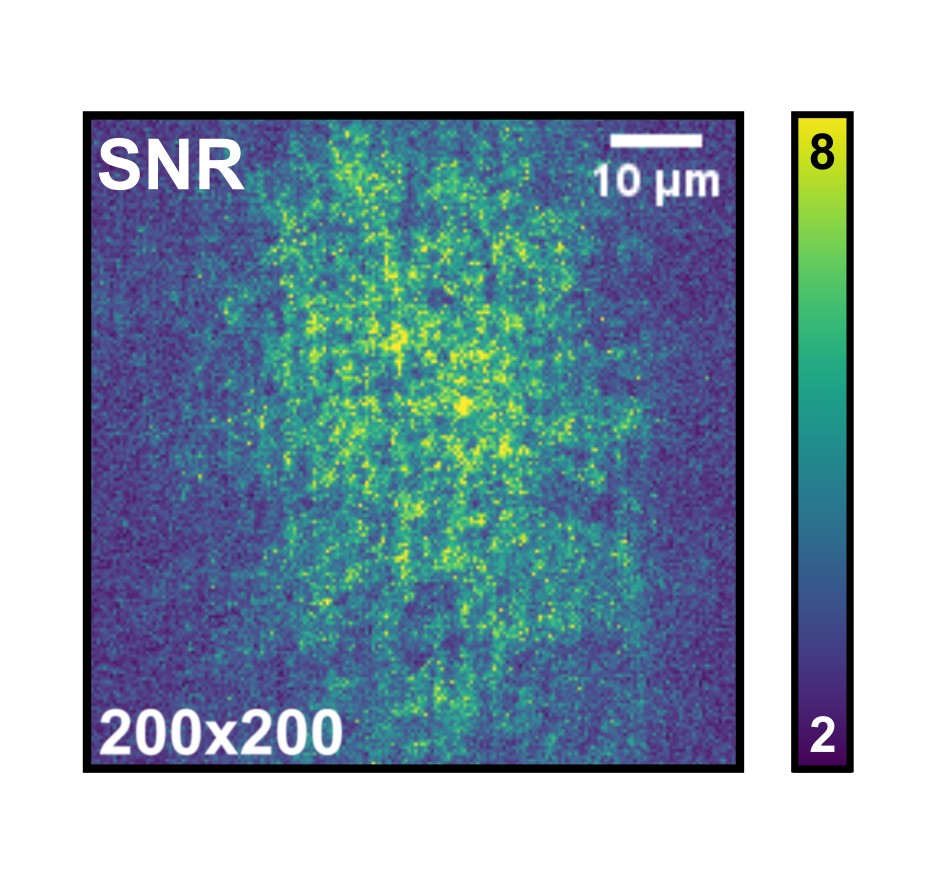


**Fig.S4:** 200$\times$200 color map of the SNR distribution at T=4$\times$𝛿t, obtained from a plain area of the USAF reflective target.

# Section S8. WAVEFORM ANALYSIS

In all the experiments presented in this work, the APD recorded signals for 100 ms. Then, during post-processing the time traces were cut in shorter time windows T. From these voltage traces, the analyzed images were reconstructed by extracting the amplitudes of the spectral components of interest. To do this, two different approaches were implemented: virtual lock-in detection^3^ and direct readout of the amplitude of the maximum peaks of the Fourier transform. The choice between the two methods depended on the time duration of the signal considered and the computational time. The steps required to extract the amplitude of the frequency components are reported in the following. In particular, for virtual lock-in detection, they are:

1. Computing the FFT of V(t)∙sin(2𝜋∙F_i,j_∙t) and V(t)∙cos(2𝜋∙F_i,j_∙t), and then the absolute value of the results
2. From the two FFT, setting to zero all the points of the array that have a non-zero frequency
3. Calculating the absolute and then computing the inverse FFT (FFT^-1^) of the two arrays obtained
4. Averaging the results to obtain two scalars A_i,j_ and B_i,j_
5. Determining the intensity of the pixel by getting the amplitude m_i,j_ as:

$m_{i,j}=\frac{\sqrt{A_{i,j}^{2}+B_{i,j}^{2}}}{2}$ (s36)

While the steps necessary for the amplitude readout from the maximum peaks are:

1. Applying a Hamming window (as defined in the Python library NumPy^4^ to the signal V(t)
2. Adding data points with values equal to zero at the end of the waveform. The total length of the appended array was eight times longer than the length of V(t). Steps 1-2 were made to reduce the effects of scalloping^5^, *i.e.* the loss of intensity in the Fourier spectral peaks described in Temporal performance.
3. Computing the FFT and the absolute value of the result
4. Taking a window of size 𝛿F centered around the expected beat frequency, and finding the maximum intensity
5. Using this value to estimate the intensity of the pixel

Let us consider now the integration time window T in terms of multiples of the minimum acquisition time 𝛿t (Fig.S5a). The FFT of a signal integrated for T=𝛿t has a frequency sampling df that is just enough to sample the frequencies that are at distance 𝛿F (top plots in Fig.S5b). Instead, for an increased integration time window, the frequency resolution increases (df<𝛿F) and the frequency components of interest (light blue) appear well-separated (middle and bottom plots in Fig.S5b). Even if schematically simplified, it is noteworthy that the amplitude of the frequency components increases by decreasing df, as expected by the FT sampling properties. As one can observe from the dashed line in Fig.S5b, when the frequency resolution is not high enough (top plot), local maxima are hardly distinguishable. In particular, the maximum detection performs very poorly when the acquisition time is T<4$\times$𝛿t. On the other hand, lock-in detection works well also in these cases.

However, virtual lock-in detection is very computationally expensive. Indeed, it requires doing N^2^ times both an FFT and an FFT^-1^ of an array of length n. On the contrary, the maximum localization requires only N^2^ reading steps of an array of a much smaller size, which makes this method much faster. Then, for a fixed sampling rate, the number of sampled points n is related to the integration time window T, which increases with the number of pixels. In conclusion, the virtual lock-in becomes computationally very expensive for some of the configurations analyzed. Thus, the maximum localization is preferentially used in this work when integrating for T≥4$\times$𝛿t. Instead, for the configuration analyzed for T<4$\times$𝛿t the virtual lock-in is used with the mentioned benefits.

To demonstrate that the two approaches are equivalent, an image of a plain mirror of 50$\times$50 pixels was reconstructed with the two methods. Once normalized, the root mean squared deviation was evaluated as:

$RMSD=\sqrt{\frac{1}{N^{2}}\sum_{p=1}^{N^{2}} {(m_{p}^{L}-m_{p}^{M})}^{2}}$ (s37)

where p is the index that runs over the pixels, and m^L^_p_ and m^M^_p_ are the pixel intensities calculated with the virtual lock-in and by reading the maximum, respectively. We obtained a value RMSD=0.033±0.002, where the value and the error were evaluated over 5 repeated measurements.


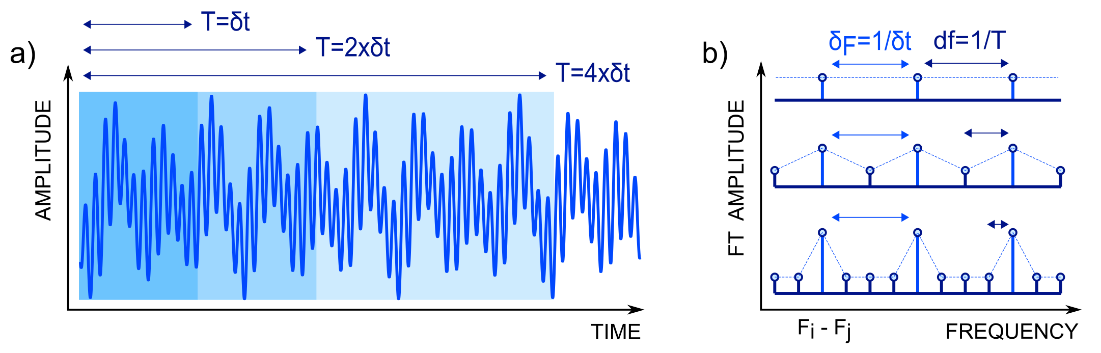


**Fig.S5**: **a)** Plot of schematic example of an acquired signal in time. The waveform is cut in subwindows of different lengths, multiples of 𝛿t (T=𝛿t, T=2$\times$𝛿t, T=4$\times$𝛿t); T: integration time window; 𝛿t: minimum acquisition time. **b)** Examples of the absolute value of a FFT of a signal sampled with three different sampling resolutions (df=1/𝛿t, df=1/(2$\times$𝛿t), df=1/(4$\times$𝛿t). The light blue peaks indicate the frequency component of interest F_i,j_=F_i_-F_j_. The largest distance between the closest beat frequencies 𝛿F remains fixed with the determined pixel configuration.

# Section S9. SNR EVALUATION

The SNR in the manuscript was calculated from 10 sets of 50$\times$50 images of a plain mirror. Each set contained images with increasing exposure time, taken as an integer multiple of the minimum integration time (from 4$\times$𝛿t up to 68$\times$𝛿t). For each exposure time, we calculated the signal as the image mean grey value and the noise as the standard deviation of the difference between two images (scaled by a factor $\sqrt{2}$). In detail, we evaluated all the possible dataset permutations, obtaining 90 values of SNR. For each given integration time, the average and the standard deviation of these 90 values return in Fig.2c,d the data points and the error bars, respectively.

The SNR map presented in Fig.S4 was calculated from 20 plain mirror images of 200$\times$200 pixels, with exposure time T=4$\times$𝛿t. From these, we obtained an average image and a standard deviation image, and then we divided them to obtain an SNR image.

# REFERENCES

(1) Niven, I.; Zuckerman, H.; Montgomery, H. An Introduction to the Theory of Numbers. **1991**.

(2) Ben-Yosef, N.; Sirat, G. Signal-to-Noise Ratio in a Frequency Multiplexing Imager. *IEEE J Quantum Electron* **1983**, *19* (12), 1741–1742.

(3) Scofield, J. H. Frequency‐domain Description of a Lock‐in Amplifier. *Am J Phys* **1994**, *62* (2), 129–133.

(4) Harris, C. R.; et al. Array Programming with NumPy. *Nature* **2020**, *585* (7825), 357–362. .

(5) Lyons, R.; Turner, C. Reducing FFT Scalloping Loss Errors Without Multiplication, *IEEE Signal Processing Magazine* **2011**, 28, 112-116
